# Supplementary material for: Patterns of Intron Gain and Loss in Fungi
Source: PLoS Biol. 2004 Nov 30;2(12):e422. doi: 10.1371/journal.pbio.0020422 (PMC532390; doi:10.1371/journal.pbio.0020422)
Supplement: Table S1 — Also available at http://genes.mit.edu/NielsenEtAl/. (4.3 MB ZIP). [file pbio.0020422.st001.zip › NielsenEtAl/html/811.html]

AN6690.1.NCU08278.1.MG06141.1.FG06222.1


```
 CLUSTAL W (1.82) Multiple Sequence Alignments - Introns Inserted


Sequence 1: MG06141.1	420 aa
Sequence 2: FG06222.1	406 aa
Sequence 3: NCU08278.1	431 aa
Sequence 4: AN6690.1	325 aa
Alignment Length: 441 aa
Number Identitical Residues: 182 aa
Alignment Score (without introns) 8879


MG06141.1 	MSLG-------------ATVPG--SESAHN--LTFSPASPDHARPRTGSNYSAETTLGGN
NCU08278.1	MVLGFEAASTHPLHPTASTSTSTSTIAPSSSPSSLSPTAVADHGLTHQQGFGTITEKDSP
FG06222.1 	MPLGLQQQDPDLAFAQSTTTSGGSTTAASD--FAQSPEHRHAAWTGRRSESRSSASLAKE
AN6690.1  	------------------------------------------------------------
          	                                                            

MG06141.1 	HNMSPNNGEFEA--RPPYLH0SMLAGGLGGTCGDMLMHSLD~TVKTRQQGDPHIPPKYTS
NCU08278.1	RNMTPDDGDFER--RPPLLQ0CMLAGGLGGTSGDMLMHSLD~TVKTRQQGDPHIPPRYTS
FG06222.1 	QNMTAQDVDVEAEGRPPYLH0AMIAGGIGGSTGDLLMHSLD~TVKTRQQGDPHVPSRYTS
AN6690.1  	--------------------~-MLAGGIG-----------D1TVKTRQQGDPHIPPRYTS
          	                      *:***:*           * ***********:*.:***

MG06141.1 	LGSSYYTIFRQEGIRRGLYGGWLPALCGSFPGTVLFFGTYEFSKRHMLDWGVQSHIAYLA
NCU08278.1	LGSSYYTIFRQEGIRRGLYGGWLPALLGSFPGTVLFFGTYEYSKRHMLDYGVQPHVAYLL
FG06222.1 	LGQSYYTIWRQEGIRRGLYGGWIPALGGSFPGTVMFFGTYEWSKRFLLDHGVQQHISYLA
AN6690.1  	MTSSYATIYRQEGLLRGLYGGVTPALMGSFPGTLIFFGVYEFTKRKMLDAGINANVAYLS
          	: .** **:****: ******  *** ******::***.**::** :** *:: :::** 

MG06141.1 	SG1FFGDLAACIVYVPSEVLKTRLQLQGRYQNPYFNSGYNYKGTVDAARTIVRQEGFSAL
NCU08278.1	SG1FLGDFAASVVYVPSEVLKTRLQLQGRYNNPYFASGYNYKGMTDAARTIVRREGFSAL
FG06222.1 	AG1FLGDLAASIVYVPSEVLKTRLQLQGRYNNPHFVSGYNYRGTIDAARTIVRLEGASAL
AN6690.1  	GG1FFADLAASVVYVPSEVAKTRLQLQGRYNNPYFKSGYNYRSTADAFRTIVRQEGFSAL
          	.* *:.*:**.:******* **********:**:* *****:.  ** ***** ** ***

MG06141.1 	FYGFQATLYRDLPFSALQFMFYEQNQIWARKWKQSREIGPQLEFLTGAAAGGLAGAITCP
NCU08278.1	FHGYKATLYRDMPFSALQFMFYEQAQSWAHKWMGSRDIGWQLELLTGAAAGGLAGSITCP
FG06222.1 	FYGYRATLYRDLPFSALQFMFWEQFTTWARKYKQSRDIGVSLELLTGAAAGGLAGVITCP
AN6690.1  	FYGYKATIFRDLPFSALQFAFYEQEQRIAKEWVGSRDIGLGLEIFTAVTAGGMAGVLTCP
          	*:*::**::**:******* *:**    *:::  **:**  **::*..:***:** :***

MG06141.1 	LDVVKTRLQTQLHPEA-IAPVKGESSLAAQSMLNNLKEPPKKHISEPSGNPGSSAQ-PQK
NCU08278.1	LDVVKTRLQTQVVPDP-TAPAIGTSSATTQAS-------TKDGTIKPTGTTESSTQKLQK
FG06222.1 	LDVVKTRLQTQIS-----APT------------------------EPRATKDHHAT-SQI
AN6690.1  	MDVVKTRIQTQQNPAPNSAPAPN----------------------ASVVREAKSGVQSQS
          	:******:***  . .. **. .                       .         . * 

MG06141.1 	RLISTSSPSTHTPRAGAAALETSSVLTGLKVIYRTEGIGGWFRGVGPRFVWTSVQSGCML
NCU08278.1	RLISTSSPSTHTPRPGAVTLDTSSVFTGLRIIYQTEGIAGWFRGVGPRAVWTSIQSGCML
FG06222.1 	RHISTSSPSTHRPRPGAVALDTSSVFTGLRMIYRTEGVAGWFRGVGPRGVWTFIQSGCML
AN6690.1  	RSISATAAAASPTPSGALRLDNSSFFTGLKMIYKTEGIGGWFRGVGPRGVWTSIQSGTML
          	* **:::.::  . .**  *:.**.:***::**:***:.********* *** :*** **

MG06141.1 	FLYQAVLRQLETYMPIEKPELAST
NCU08278.1	FLYQAILRQLETRMPGEQERTEVA
FG06222.1 	FLYQRLLRQLEDFMPNELKEM---
AN6690.1  	VLYQYLLKQFEAFGEAEDTNPL--
          	.*** :*:*:*     *  .
```
